# Precise characterization of a corridor-shaped structure in Khufu’s Pyramid by observation of cosmic-ray muons

**DOI:** 10.1038/s41467-023-36351-0

**Published:** 2023-03-02

**Authors:** Sébastien Procureur, Kunihiro Morishima, Mitsuaki Kuno, Yuta Manabe, Nobuko Kitagawa, Akira Nishio, Hector Gomez, David Attié, Ami Sakakibara, Kotaro Hikata, Masaki Moto, Irakli Mandjavidze, Patrick Magnier, Marion Lehuraux, Théophile Benoit, Denis Calvet, Xavier Coppolani, Mariam Kebbiri, Philippe Mas, Hany Helal, Mehdi Tayoubi, Benoit Marini, Nicolas Serikoff, Hamada Anwar, Vincent Steiger, Fumihiko Takasaki, Hirofumi Fujii, Kotaro Satoh, Hideyo Kodama, Kohei Hayashi, Pierre Gable, Emmanuel Guerriero, Jean-Baptiste Mouret, Tamer Elnady, Yasser Elshayeb, Mohamed Elkarmoty

**Affiliations:** 1 IRFU, CEA, Université Paris-Saclay, F-91191 Gif-sur-Yvette, France; 2 Nagoya University, 1 Furo, Chikusa, Nagoya, Aichi 464-8602 Japan; 3 PRESTO, Japan Science and Technology Agency (JST), Saitama, 332-0012 Japan; 4 Cairo University, Gamaa Street, 12613 Giza, Egypt; 5 HIP Institute, 50 rue de Rome, 75008 Paris, France; 6 Dassault Systèmes, 10 Rue Marcel Dassault, 78140 Vélizy-Villacoublay, France; 7 Whatever The Reality, 5 chemin de Picurey, 33520 Bruges, France; 8 High Energy Accelerator Research Organization (KEK), 1-1 oho, Tsukuba, Ibaraki 305-0801 Japan; 9 Emissive, 71 rue de Provence, 75009 Paris, France; 10 Université de Lorraine, CNRS, Inria, Nancy, F-54600 France; 11 Ain Shams University, Kasr el-Zaafaran, Abbasiya, Cairo, Egypt

**Keywords:** Experimental particle physics, Characterization and analytical techniques, Imaging techniques

## Abstract

Khufu’s Pyramid is one of the largest archaeological monument all over the world, which still holds many mysteries. In 2016 and 2017, the ScanPyramids team reported on several discoveries of previously unknown voids by cosmic-ray muon radiography that is a non-destructive technique ideal for the investigation of large-scale structures. Among these discoveries, a corridor-shaped structure has been observed behind the so-called Chevron zone on the North face, with a length of at least 5 meters. A dedicated study of this structure was thus necessary to better understand its function in relation with the enigmatic architectural role of this Chevron. Here we report on new measurements of excellent sensitivity obtained with nuclear emulsion films from Nagoya University and gaseous detectors from CEA, revealing a structure of about 9 m length with a transverse section of about 2.0 m by 2.0 m.

## Abstract (web summary)

Khufu’s Pyramid is one of the largest archaeological monuments in the world, and still contains unexplored voids. Here, the authors use cosmic-ray muon radiography in multiple positions to precisely characterize one of these inner structures called the North Face Corridor.

## Introduction

**Fig. 1:**
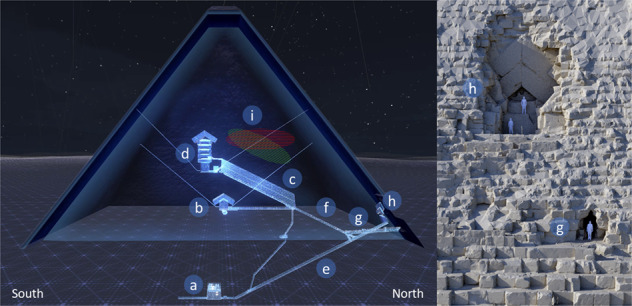
East-West cut view of the Great Pyramid and front view of the North face Chevron area. **a** Subterranean chamber, **b** queen’s chamber, **c** grand gallery, **d** king’s chamber, **e** descending corridor, **f** ascending corridor, **g** al-Ma’mun corridor, **h** north face Chevron area, **i** ScanPyramids Big Void with horizontal hypothesis (red hatching) and inclined hypothesis (green hatching) as published in November 2017^6^. All these images were obtained from a 3D modelization using dedicated laser surveys and photogrammetry data.

**Fig. 2:**
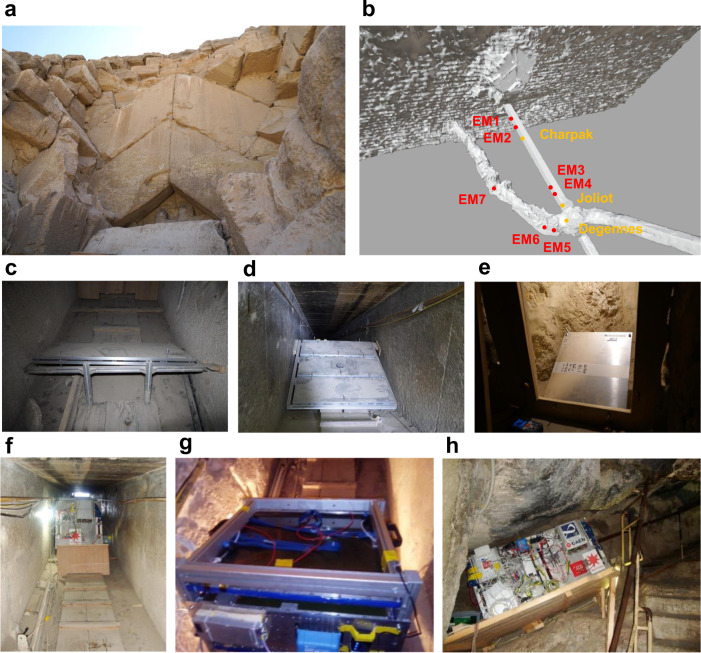
Detectors installed in the DC and in the MC. **a** The Chevron, which consists of huge gabled limestone beams, covering the original entrance to the DC on the North side of Khufu’s Pyramid. **b** 3D model and positions of the detectors from Nagoya University, indicated by red dots and of the detectors from CEA, indicated by orange dots, in the DC and in the MC. **c**–**h** The detectors. **c** shows EM3, **d** shows EM2, **e** shows EM5, **f** shows Charpak, **g** shows Joliot and **h** shows Degennes.

The Great Pyramid is one of the largest stone structures in the world, built 4500 years ago by the king Khufu, king Snefru’s son, on the Giza plateau of necropolis in Egypt. It was expected to be over 146 m high, before its smooth outer coating was stripped off by carriers in the Middle Ages as well as the capstone on the top. Today, the pyramid is 139 m high and 230 m wide, and is thought to be made of several million pieces of limestone, each about 1–2 m high. There are large internal structures in its massive stone body, connected by narrow corridors in the north-south direction at a distance of about 7 m east from the center of the pyramid (Fig. 1). They are respectively called, from the bottom to the top, the abandoned subterranean chamber (SC), the queen’s chamber (QC), the grand gallery (GG) and the king’s chamber (KC)—the only room built with granite stones including its broken sarcophagus. The passage dug by al-Ma’mun in the Middle Ages from the central axis of the pyramid to the drop stone located at the intersection of the descending corridor (DC) and the ascending corridor (AC) is called al-Ma’mun corridor (MC) and now used as the tourists entrance. A stone slab with a gabled structure called the Chevron (Figs. 1 and 2a) has been located at the top of the entrance connected to the DC made at the time the pyramid was built. This structure is slightly excavated from the surface of the pyramid and is thought to have been originally hidden inside the surface of the pyramid. Khufu’s Pyramid is the first pyramid, in the history, that uses a Chevron technique to cover internal structures and prevent them from collapsing. We can find Chevron on the North Face, in the queen’s chamber ceiling and above the king’s chamber. The construction process of the oldest of the seven wonders of the ancient world is one of the most important archaeological mysteries. Any discovery of previously unknown internal structures could contribute to the knowledge on the construction of this Pyramid.

We have investigated the internal structure of Khufu’s Pyramid using a technique based on the observation of cosmic-ray muons. Matter naturally absorbs a fraction of these muons coming from the atmosphere, this fraction is determined by the thickness and by density of the probed object. The measurement of the muon flux in a given direction then provides a direct estimate of the mean density in that direction. From such measurements, 2D density maps can be obtained revealing the inner structure of an object. This technique was first used by Alvarez et al.^1^ for the search of hidden chambers in Khafre’s Pyramid and has also been used for investigations of volcanoes^2^ and nuclear reactors^3,4^ among others^5^. In 2017 we discovered a large cavity named ScanPyramids Big Void (SP-BV) using three types of muon detectors.

In this paper, we report on the first precise analysis of the void found with cosmic-ray muon radiography behind the North Face Chevron and named the ScanPyramids North Face Corridor (NFC). Its shape and location are accurately evaluated by focusing on the 7 following parameters: its 3 dimensions (width *W*, height *H*, length *L*), its position (North-South position *X*, East-West position *Y*, altitude *Z*) and its slope *α*. While a potential, future exploration will primarily require a precise determination of *Y* and *Z*, most of these parameters are also important to understand the role of the Chevron.

## Results

### Measurement by Nagoya University

We used nuclear emulsion films in the DC to investigate the region behind the Chevron (Fig. 2a). Nuclear emulsion film is a photographic film-type particle detector that can record the trajectory of a charged particle without electric power supply. It is particularly suitable in narrow spaces like the DC because it is compact and lightweight. A nuclear emulsion film^6^ with an active area of 25 × 30 cm^2^ was made at Nagoya University by coating nuclear emulsion gel to both sides of transparent plastic base. After the observation in the DC, nuclear emulsion films were developed in a darkroom at the Grand Egyptian Museum Conservation Center (GEM-CC) at Cairo, and then transported to Nagoya University to be analyzed using an automated nuclear emulsion scanning system called Hyper Track Selector (HTS)^7^. By analysing three nuclear emulsion films with measurement period of 67 days, we reported on the discovery of NFC by detecting an unexpected muon excess in 2016^8^. It seemed to be an elongated void of at least 5 m length oriented in the North-South direction above the DC.

From 2016 to 2019, nuclear emulsion films were installed at several positions in the DC and MC toward the NFC to reveal the three-dimensional shape, location and inclination of the NFC as shown in Fig. 2. Multi-point observations from DC can provide high-resolution images of the NFC from a short distance just below it, which can be combined and analyzed to reveal the configuration and location of the NFC. Multi-point observations from the MC can reveal the inclination, location, and vertical layout of the NFC from the side. Both observations are thus complementary.

### Analysis of detectors installed in the descending corridor by Nagoya University

For the observations in the DC, detectors consisting of an aluminum honeycomb plate mounting three nuclear emulsion films were assembled. Six sets of detectors, respectively named EM1, EM2N, EM2C, EM2S, EM3, and EM4, were covered by wooden boxes for protection and installed at four locations in the DC (Fig. 2). The nuclear emulsion films were replaced periodically every few months to avoid performance degradation^9^. In this analysis, data from February to October 2019 were used (Methods). Observed muons were analyzed by summing different observation periods, 172 days for EM1, 211 days for EM2 and EM3, and 79 days for EM4. The number of muon tracks recognized within the angular range of ∣$\tan {\theta }_{x,y}$∣ ≦ 1.0 was 9.48 × 10^7^ tracks for EM1, 9.39 × 10^7^ tracks for EM2N, 2.90 × 10^7^ tracks for EM3 and 9.87 × 10^6^ tracks for EM4 (Methods). The observed muon flux distributions were compared with expectations from the Geant4^10–12^-based simulation framework containing the 3D model around the Chevron and the known internal structures with the muon flux formula given by Guan^13^ (Fig. 2b and Methods). Simulations were performed for EM1 and EM2, each equivalent to an acquisition of 500 days, and for EM3 and EM4, each equivalent to 1000 days, so that statistical fluctuations from the simulations are negligible.

**Fig. 3:**
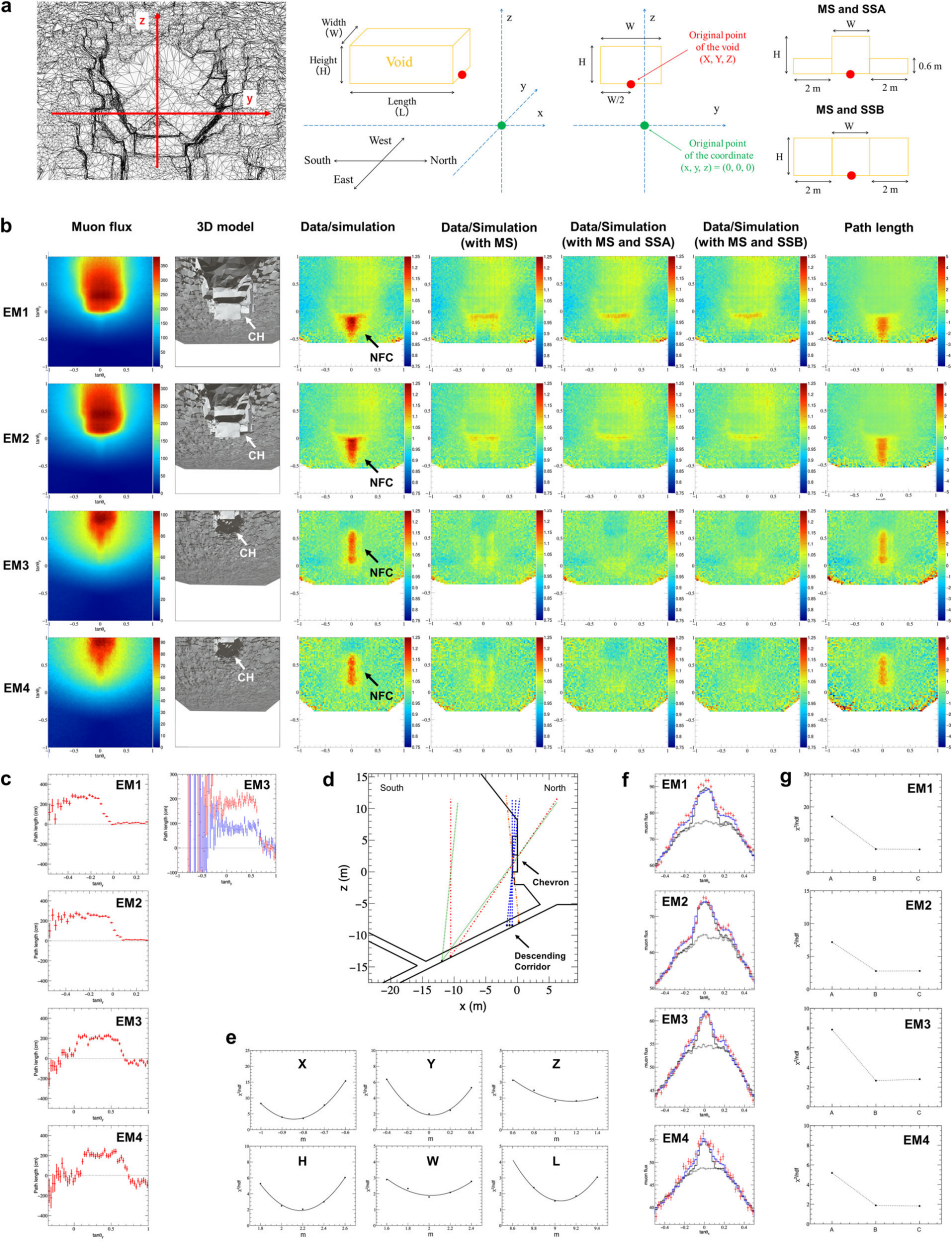
Results of the analysis of the nuclear emulsion films installed in the descending corridor. **a** The left figure shows the 3D model of Chevron and its origin. Center panels show the definition of the coordinate system and the origin of the rectangular cuboid which was defined as its North extremity along *x*, its center along *y*, and its bottom along *z*. Right panels show the set up of the main structure (MS) and sub-structures designated A (SSA), B (SSB). CH denotes the Chevron. **b** Two-dimensional angular distribution for EM1 to EM4. From left to right: observed muon flux (tracks/cm^2^/day/sr), 3D model, ratio of muon flux of the observed data to simulation, with MS, with MS and SSA, with MS and SSB, and path length with difference between the data and simulation. The resolution is $\tan \theta$ = 0.025. **c** Histograms of path length with difference between the data and simulation in the range of 0.000 ≦ $\tan {\theta }_{x}$ < 0.025 in the axial direction of $\tan {\theta }_{y}$ corresponding to the North-South direction. **d** Region of existence of the NFC based on path length. **e** *χ*^2^ analysis for the evaluation of parameters presenting location and shape of the NFC. The horizontal axis shows the values of evaluated parameters. The vertical axis is the value of reduced *χ*^2^ obtained by the comparison of the data and simulation. **f** Histograms of muon flux in the range of −0.250 ≦ $\tan {\theta }_{y}$ < −0.225 for EM1 and EM2, 0.200 ≦ $\tan {\theta }_{y}$ < 0.225 for EM3 and EM4, respectively. The data with statistical error of 1 *σ* (standard deviation) are shown in red. The gray dashed line is the simulation without the inner structures, the black solid line is the simulation with the DC and MS, and the blue solid line is the simulation with the DC, MS, and SSA. **g** Results of the *χ*^2^ analysis for the case where the MS and sub-structures are added to known structures. A, B, and C denote the case where MS, MS and SSA, MS and SSB are added, respectively.

By comparing the observations and normalized simulations, muon excess regions due to the NFC were clearly detected from all detectors (EM1-EM4) and the surrounding regions were in good agreement except around the boundaries of the 3D model (Fig. 3b). In this analysis, the angular zone outside the region of interest and around the edge of the 3D model was excluded.

Data normalization was performed to suppress the effects of systematic errors due to the detection efficiency of muons on nuclear emulsion film depending on the observation period and scanning conditions, and to simulation conditions.

Data normalization was performed with a simulation in a region outside the NFC by matching the mean values of the data to simulations for each slice in $\tan {\theta }_{y}$ divided by a width of $\tan \theta$ = 0.025. The muon excess significance estimated from statistical errors was well over 10 *σ* (Fig. 3f).

In order to localize and estimate the dimensions of the NFC, which was assumed to be a rectangular cuboid, simulations assuming a void of various dimensions (width, height and length) and positions were performed and compared with data as shown in Fig. 3a. In this analysis, the coordinate system and its origin chosen from a specific point on the North Face Chevron were defined, also showing the three-dimensional structure of the NFC and its origin. The 7 parameters chosen to characterize the NFC were obtained through *χ*^2^ analyses from the comparison between observation and simulations (Methods).

Based on the initial analysis of path length distributions with difference between observation and simulation for rough estimation of shape and location (Fig. 3c, d and Methods), we tentatively set the 7 parameters of dimensions (*W* = 2.0 m, *H* = 2.0 m, *L* = 9.5 m), location (*X* = −1.0 m, *Y* = 0.0 m, *Z* = 0.5 m) and slope *α* = 0° to compare the data and the corresponding simulations of each detector. The path length is defined as the total length of the material (in this case, the stones that construct the pyramid) existing in each direction from the detector. Therefore, the difference between observation and simulation in the path length will reflect the depth and shape of the NFC. As a result, a small excess of muons was observed in all data along the periphery of the main muon excess in the east-west direction (Fig. 3f). The amount of small excess is equivalent to ~0.6 m of void. Therefore, we considered the possibility that a substructure with small muon excess exists around the main structure, which is about 2 m high, and conducted the subsequent analysis taking this structure into account. By using a 2 m height for the main structure and the region where the void is located (*z* > 0, *x* < 0) as initial conditions and comparing them with various simulations, the parameters that determine the shape (*W*, *H* and *L*) and the position (*X*, *Y* and *Z*) can be obtained, assuming that the object is a single rectangular cuboid and horizontal (*α* =  0°). Each parameter was evaluated by *χ*^2^ analysis in the order of *X* by EM2, *Z*, *H*, *W*, *L*, and *Y* by EM3, by taking into account the combination of detectors that can be determined with high accuracy (Fig. 3e and Methods). The regions of evaluation of *χ*^2^ were determined according to each parameter. The main structure was evaluated by considering the influence of the sub-structure and inconsistency of entrance region after normalization.

The shape of the NFC defined as a rectangular cuboid was estimated to have a width of 2.02 ± 0.06 m, a height of 2.18 ± 0.17 m and a length of 9.06 ± 0.07 m. The NFC appears to stop at the South side and simulation confirmed that there is no structure with a cross section of more than 1 m × 1 m further on the South (Fig. 3c and Methods). The bottom plane of the NFC is located at *Z* = 0.72 ± 0.13 m which corresponds to ~20 m from the ground level. The East-West central axis of the NFC is located at *Y* = 0.03 ± 0.04 m, which is coincident with the center of the Chevron and above the DC. The distance from the North face of NFC to the North face of the Chevron is 0.84 ± 0.05 m which is about the same thickness as the massive limestone block of the chevron that can be observed from the outside, suggesting the NFC would be located just behind it.

The possibility of the existence of sub-structures around the main structure was evaluated against the observed results from the DC. Using the same method as the *χ*^2^ analysis conducted to determine the parameters of the main structure, we evaluated the reduced *χ*^2^ in the case that the sub-structures were added to both sides of the main structure. Simulations were performed for two cases of sub-structures, A: voids of 0.6 m in height and 2 m in width placed on both sides along the main structure and B: a limestone-equivalent with a density of 1.6 g/cm^3^, which corresponds to about 73% fill for a surrounding region with a density of 2.2 g/cm^3^, occupying the region of the same height as the main structure and 2 m in width placed on both sides of it (Fig. 3a). The results show that the agreement between the observed muon flux distribution and the simulation is better for EM1, EM2N, EM3, and EM4 with the two structures, since the reduced *χ*^2^ is smaller than that with the main structure only (Fig. 3g). However, these two assumptions are only a subset of the possibilities, and in the absence of constraints on the elevational extent of the substructure, it is difficult to determine whether the region contains obvious space or low-density regions with high porosity, such as debris, along with its vertical extent.

### Analysis of detectors installed in the al-Ma’mun Corridor by Nagoya University

For the observations from the MC, four detectors (EM5, EM6H, EM6T, and EM7) were installed in three narrow hollows on the wall of the MC, for a total observation period of 272 days (Fig. 2). This setup allows for observations from the sides of the NFC and the three detectors (EM5, EM6T, and EM7) were tilted so that the observation direction points towards the NFC (Methods).

In the MC, the detectors EM5, EM6T, and EM7 were installed with leaning against the wall of the narrow hollows, which may cause the detectors to change direction when they are replaced. The differences of direction of each detector were corrected with an accuracy of 0.2° or less by comparison between the angular distributions of muons obtained in each observation. Through the correction process, all observations at the same location were combined based on the first observation period from February to April 2019 and the combined angular distribution of muons were obtained for 272 days (Methods). The number of muon tracks recognized within the angular range of ∣$\tan {\theta }_{y}$∣ ≦ 1.0 was 8.69 × 10^6^ tracks for EM5, 8.76 × 10^6^ tracks for EM6H, 7.38 × 10^6^ tracks for EM6T and 1.11 × 10^7^ tracks for EM7, respectively.

**Fig. 4:**
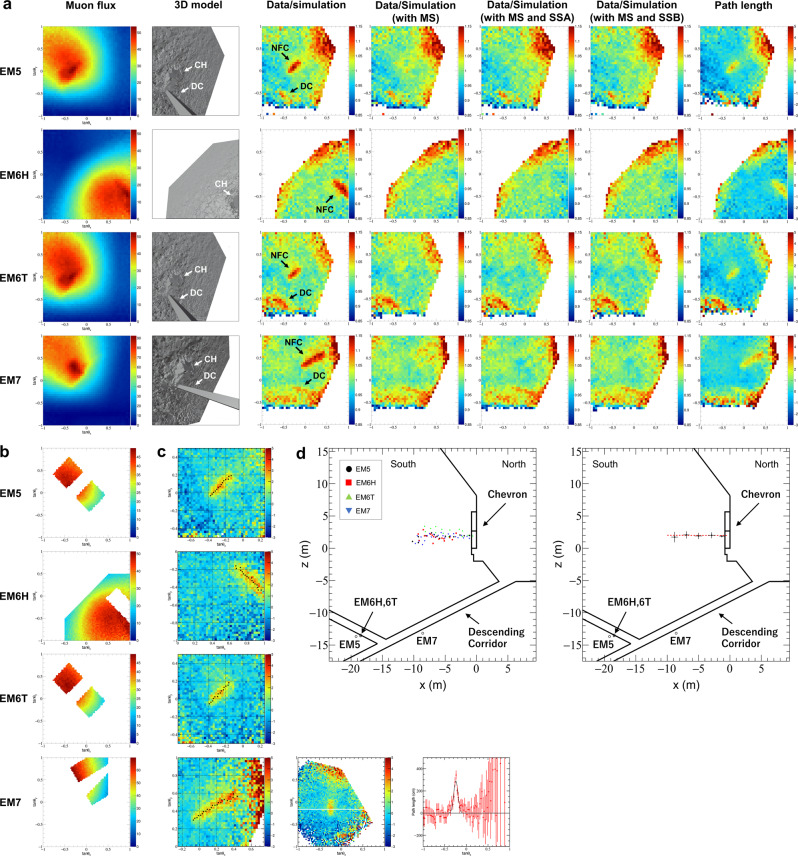
Results of the analysis of the nuclear emulsion films installed in the al-Ma’mun Corridor. **a** Two-dimensional angular distribution for EM5 to EM7. From left to right: observed muon flux (tracks/cm^2^/day/sr), 3D model, ratio of muon flux of the data to simulation, with main structure (MS), with MS and sub-structure A (SSA), with MS and sub-structure B (SSB), and path length with difference between the data and simulation. The resolution is $\tan \theta$ = 0.050. CH denotes the Chevron. **b** The area used for normalization. **c** Left panels show two dimensional angular distribution with enlarged area including NFC of path length difference between the data and simulation with the resolution of $\tan \theta$ = 0.025. The black dots are the direction to the center of the NFC, obtained by taking a cross section every $\tan \theta$ = 0.025 and fitting it (Methods). Center panel shows an example of path length difference obtained by EM5, and the coordinate system is rotated to take the cross section for fitting, which is perpendicular to the longitudinal direction (North-South direction) of the NFC. Right panel shows an example of the fitting to a cross section taken at $\tan {\theta }_{y}$ = −0.1625 after rotation, which is indicated by the white line shown in center panel, where the horizontal axis is $\tan {\theta }_{x}$ after rotation and the vertical axis is the difference in path length. **d** Location of the NFC. Left panel shows the intersection of a vertical plane passing through the central axis of the DC and a line extending towards the plane on the direction of the central value of the histogram fitted starting from the detector position. Right panel shows the result of averaging the projection points obtained by all detectors in the height direction with a width of 2 m in the *x* (North-South) direction. The error bars are the standard deviations of the averaged projection points in the *x* and *y* directions. The red dotted line is the approximate line obtained by linear approximation of the projection results in the range of −2 to −8 m.

Figure 4a shows the angular distribution of the observed muon flux divided by the flux obtained from the Monte Carlo simulation based on Geant4, using the determined azimuthal angle from the analysis and the 3D model excluding the DC structure. The normalization of the simulation to the observation in terms of muon flux was conducted in the same way as for the analysis of the DC in the area outside of the NFC and not including the Chevron region (Fig. 4b). The simulation corresponding to an observation period of 400 days was individually performed for EM5, EM6H, EM6T, and EM7.

By comparing the data with the corresponding simulations, the NFC was clearly detected from all detectors, with a muon excess significance well over 10 *σ*, while the surrounding regions were in good agreement except around the boundaries of the 3D model and for low elevation angle. In addition the DC, which has a cross section of ~1 m × 1 m, is also visible for EM5, EM6T, and EM7 (Fig. 4a).

The direction pointing to the center of the NFC was determined from each detector. Its direction was defined as the center value of the fitting with Gaussian function of the cross-sectional distribution of the region corresponding to the NFC by determining path length distribution from the muon flux distribution (Fig. 4c).

**Fig. 5:**
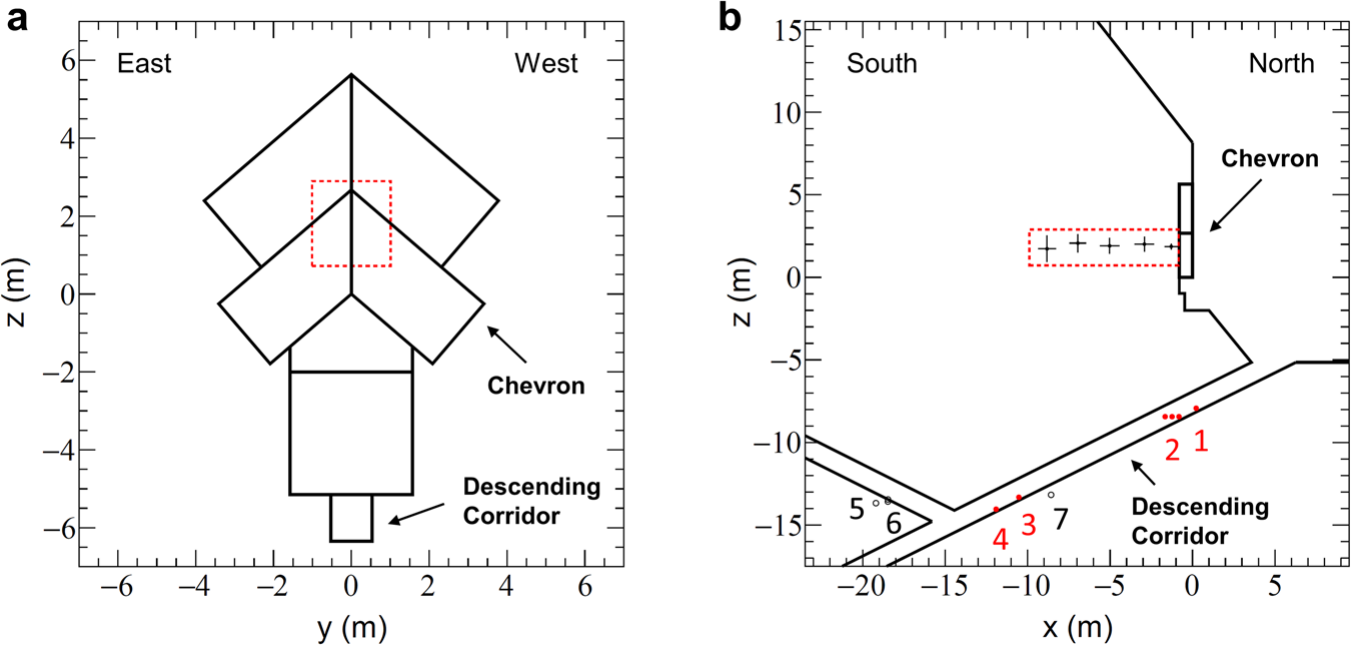
Results of the analysis of the nuclear emulsion films by Nagoya University. The location and shape of the NFC are shown in cross-sectional views of the analysis area including Chevron from the North (**a**) and East (**b**) sides. Locations of EM1 to EM4 detectors installed in the DC and obtained results are shown in red, and EM5 to EM7 installed in the MC are shown in black, respectively in **b**. The red dotted line is the projection of the location and shape of the NFC defined as a rectangular cuboid achieved by EM1 to EM4, on planes. Black dots are the projection points in the height obtained by detectors (EM5 to EM7) on planes and error bars, which is the standard deviations of the averaged projection points in the *x* and *z* directions as shown in Fig. 4d.

Since the observations from the DC confirmed that the NFC was located just above the DC, the direction of the NFC from detectors at the MC was projected onto a vertical plane passing through the center of the DC (Fig. 4d and Methods). By analyzing the spatial distribution, the NFC center in *z* is evaluated at 2.0 ± 0.5 m, the length is ~10 m from behind the Chevron and the slope *α* is −0.3 ± 1.5° (Fig. 5b and Methods). Comparing the data with simulations including different rectangular cuboids with cross sections varying by 0.5 m, the best agreement was obtained for cross sections of 1.5–2.0 m in height and 2.0–2.5 m in width. Since this observation was conducted from the side of the NFC, the possibility of a low-density region spreading vertically, which is not easy to determine from a detector in the DC, was clearly eliminated. The results from the MC are consistent with the cross-section obtained from the DC. In the case where the substructure is attached to the main structure, they seem to overlap in the East-West direction when observed from the MC, so the width of the void from observations might be larger than that of the main structure alone. The agreement between the observations and the simulations with and without the substructures for the main structure with a cross section of 2 m × 2 m was evaluated, and the results were the same or better with the substructures (Fig. 4a).

### CEA analysis

The telescopes designed and operated by the CEA are made of micro-pattern gaseous detectors called multiplexed Micromegas. Their principles and performance are described in ref. ^14^. Contrary to emulsions, such telescopes are less compact and require electricity, though with limited power (~50 W). However the data can be processed online with a single mini-PC, allowing for fast results. Moreover, unexpected displacements during the acquisition can also be handled without any loss of data. The measurement campaign started in October 2019 with the installation of three telescopes in the DC and on a dedicated wooden platform built at the intersection between the DC and the AC (Fig. 2b, f–h). The middle telescope (called *Joliot*) had an active area of 50 × 50 cm^2^, while the other two (*Degennes* and *Charpak*) were made of two parts, resulting in a surface of 100 × 50 cm^2^ each. The excellent spatial resolution of the detectors, of the order of 200 μm, yields an angular resolution around 1 mrad only yet with very compact instruments to fit in the DC. Their positions were carefully chosen to perform a precise triangulation, Degennes telescope being also positioned to probe the potential end of the NFC or a hypothetical connexion with the Big Void. The 3 telescopes were flushed using a single 20 L, pressurized bottle of non-flamable gas (Ar-iC_4_H_10_-CF_4_ 95-2-3) as for the previous campaigns. Thanks to an optimized gas circuit with filtering and recirculating units^15^, the total gas consumption could be reduced down to 0.5 L/h in steady mode. Ethernet cables were installed in the DC to connect the 3 telescopes to a 4G router with its antenna placed just below the Chevron. This router ensured the remote control of the instruments from CEA in France and the transfer of the reconstructed muon data.

**Fig. 6:**
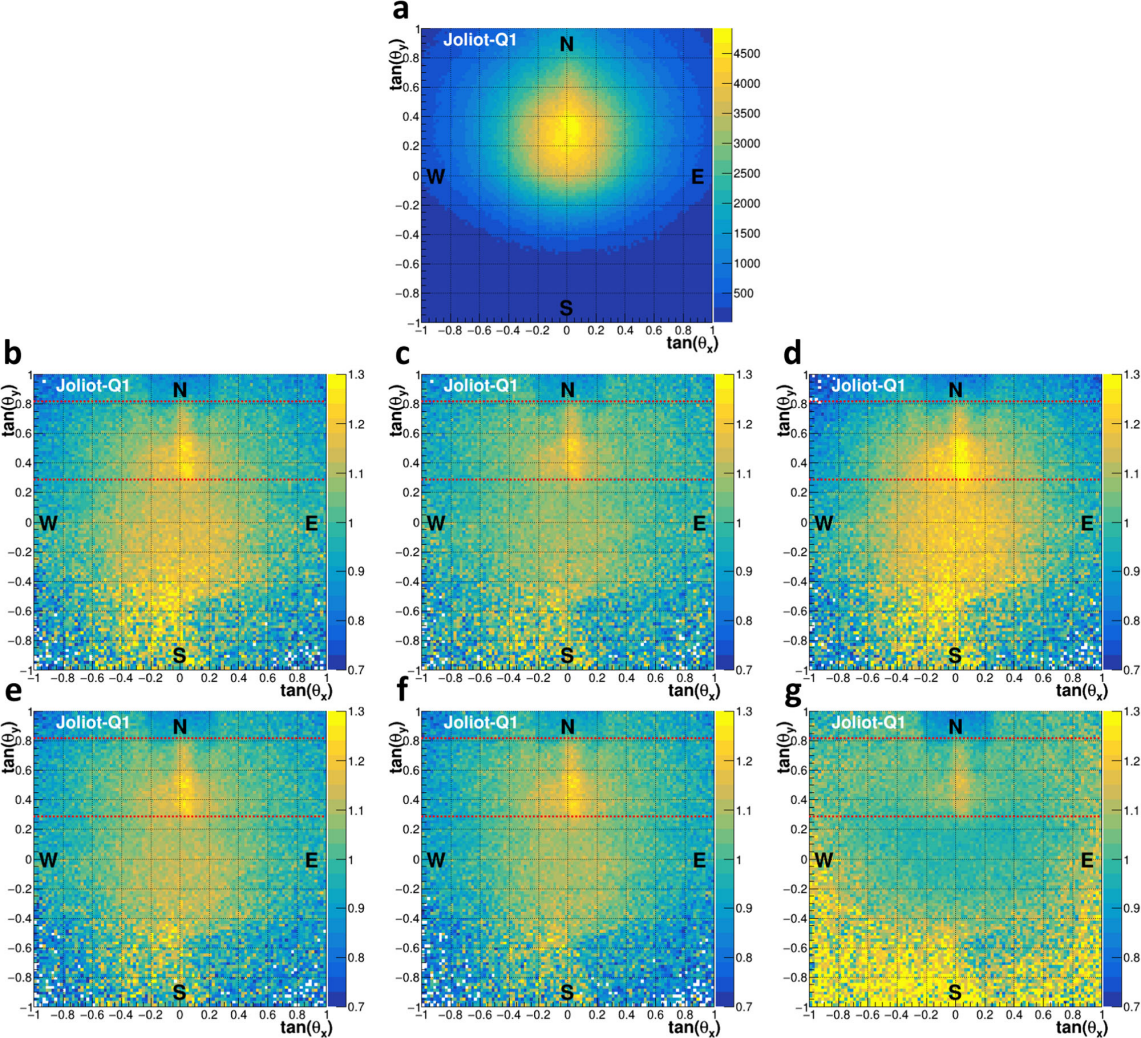
Effect of the muon parametrization on the comparison between data and simulation. **a** Muography of the Joliot instrument. **b**–**g** Ratio between Joliot muography and Geant4 simulations of the known structures, using parametrizations from Tang (**b**), Reyna (**c**), Matsuno (**d**), Bogdanova (**e**), Guan (**f**), and Shukla (**g**).

All in all, the telescopes acquired about 140 days of stable data (Methods), collecting more than 116 millions of muons. The corresponding five instruments (1 + 2 + 2) were treated independently in the analysis. The 2D raw muographies were obtained like in ref. ^16^ from the angular distribution (tan(*θ*_*x*_), tan(*θ*_*y*_)) of the reconstructed muons (see for example Fig. 6a). These data were compared with Geant4^10–12^-based simulations of the pyramid (Methods) containing all the known structures. To do this, a precise knowledge of the position and orientation of each telescope is required. The positions were measured in situ with a centimeter resolution and validated with the 3D model for which a 10 cm accuracy was considered. The orientations (Euler angles) were first derived with a 1° accuracy using electronic probes from the Yoctopuce company, and later refined down to 0.2–0.4° by a *χ*^2^ analysis of the muon data (Methods).

An important aspect of the simulation is the choice of the cosmic muon parametrization. Six of them were investigated, as shown in Fig. 6b–g. As independently obtained by Nagoya analysis, the Guan parametrization^13^ was found to provide the best agreement with CEA data in terms of relative and absolute muon flux, though Bogdanova^17^, Reyna^18^, and Tang^19^ were also close. Matsuno^20^ and Shukla^21^ deviated significantly, but it’s worth mentioning that the former was derived for nearly horizontal muons while the latter provided parameters at a given muon direction, a situation not adapted to our large acceptance instruments. Overall, data and simulation agree within 15% with the selected parametrization except in the direction of the summit, a region with poor statistics and out of interest for the current analysis. Data also exhibit a mean deficit of muons in the Chevron zone due to the higher density of the stones used in this area. Starting from the Chevron, a very clear excess appears as a vertical line. This excess is visible on each of the 5 muographies and corresponds to the NFC, with a statistical significance largely above 10 *σ*. The excess yields about 15 muons/h for Joliot, Degennes-1 and Degennes-2, and about 60 muons/h for Charpak-1 and Charpak-2. By the end of November 2019, the global shape of the NFC as well as its extremities were thus well visible. Last but not least, the NFC excess is visible with all the 6 parametrizations (Fig. 6b–g) and therefore cannot be attributed to any bias from a particular one.

**Fig. 7:**
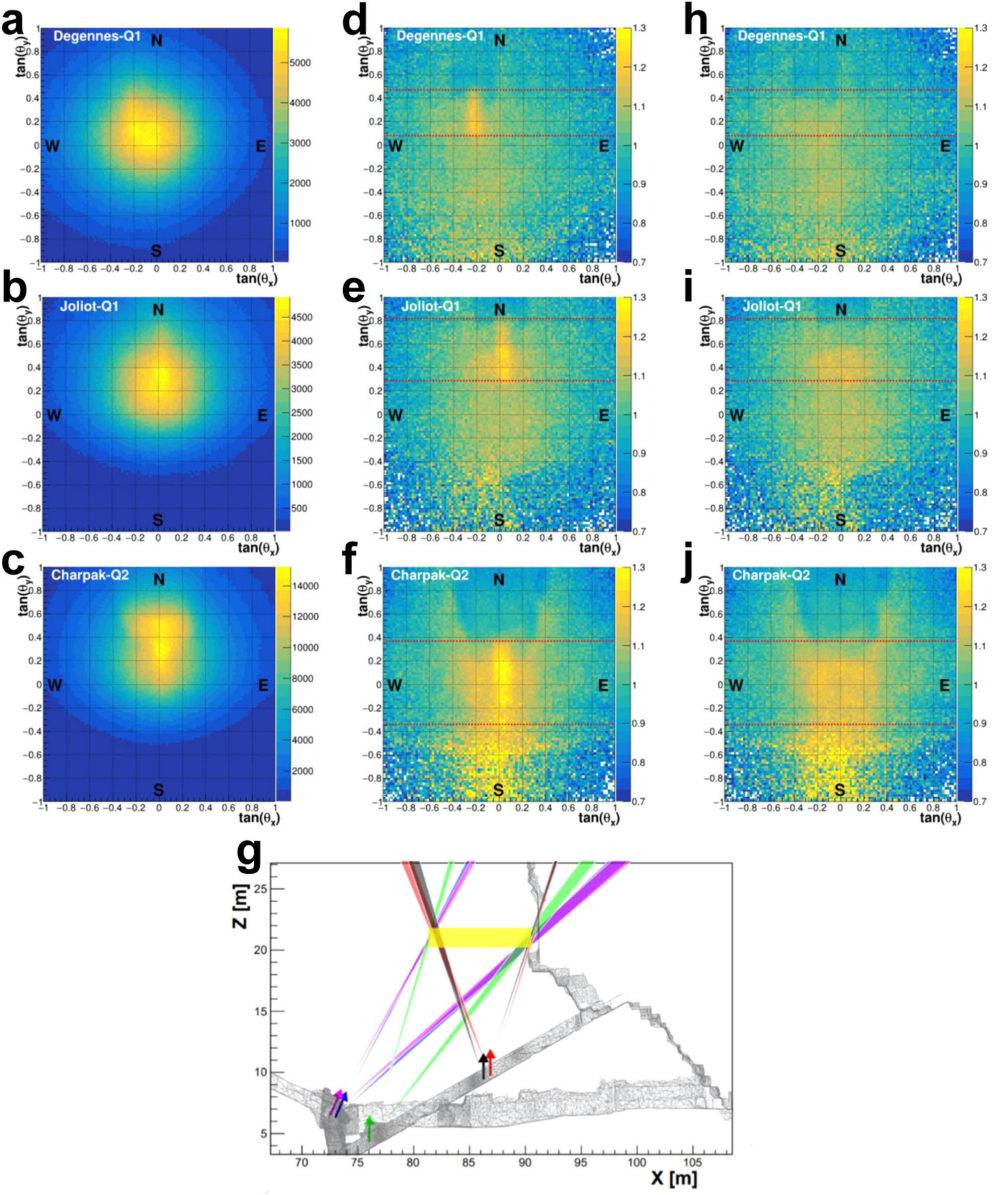
NFC signal and triangulation from CEA data. **a**–**c** Raw muographies obtained with Degennes-1, Joliot and Charpak-2. **d**–**f** Ratio between the data and the simulation for these three instruments, showing good overall agreement except in the summit direction where the statistics is very low. The horizontal, dashed red lines indicate the limit of the NFC obtained by slicing each image. **g** Triangulation of the NFC using the five instruments, where each cone represents the extremity of the NFC found in one data set (i.e., the directions defined by the red lines in plots (**d**–**f**)). The width of each cone represents the uncertainty of its direction, i.e., from 0.5 to 1.5°. The arrows show the orientation of each instrument. The yellow rectangle represents the position and size of the NFC as determined from the CEA analysis. **h**–**j** Same ratio as in **d**–**f**, obtained with a simulation containing a void representing the NFC, whose dimensions are described in the text. The excess seen in **d**–**f** has largely disappeared.

From the muography images and the division with simulation (Fig. 7a–f), each instrument can provide the extremities of the NFC. To do so, 1D histograms were obtained from thin (0.02) horizontal slices on the 2D plots to determine the appearance and disappearance of the NFC muon excess along the $\tan {\theta }_{y}$ axis like in ref. ^16^ (Methods). These extremities are represented by red dotted lines in Fig. 7d–f. Within statistical errors, these extremities form cones in the x-z plane, originating from each instrument as reported in Fig. 7g.

**Fig. 8:**
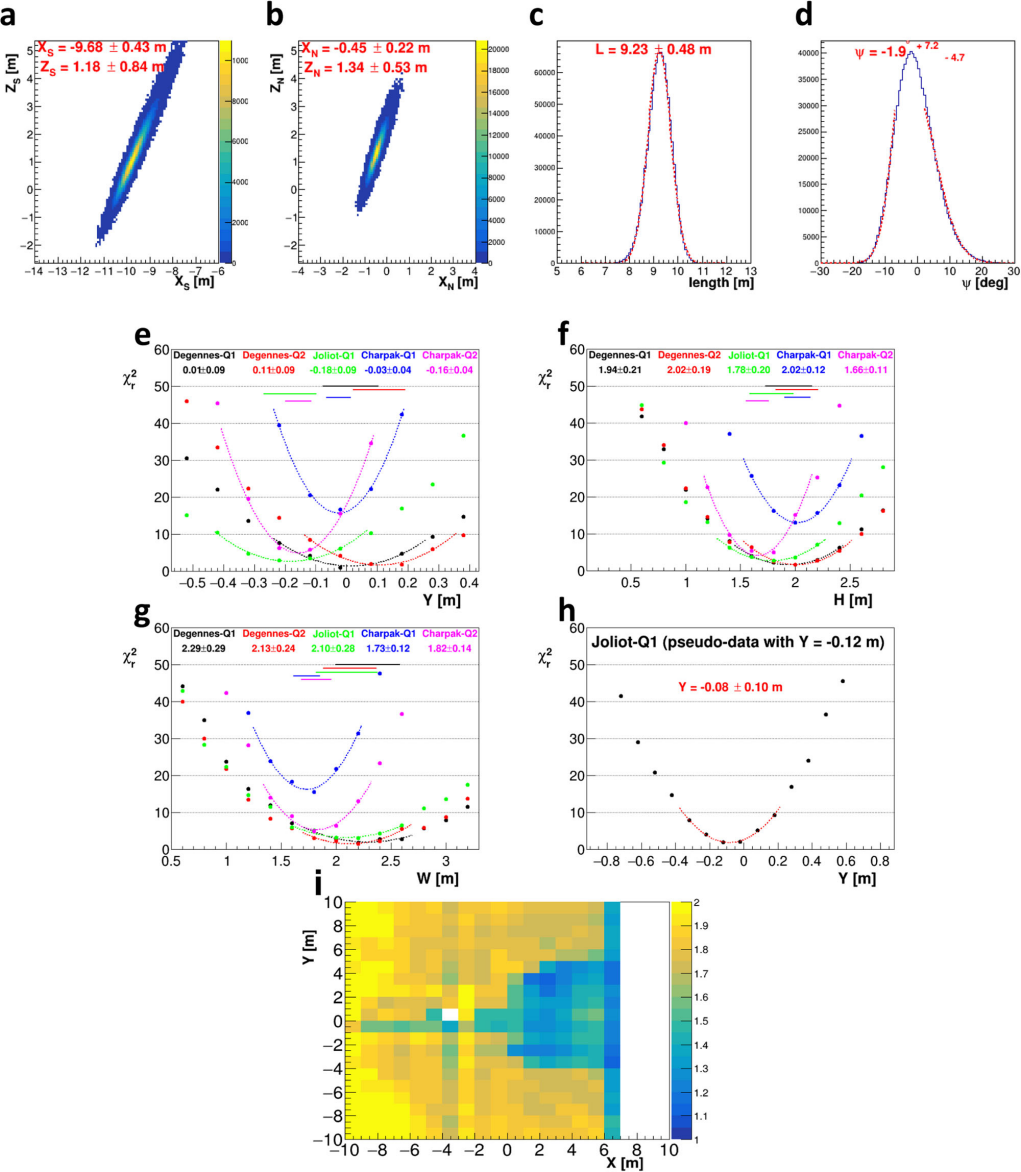
Determination of NFC characteristics from CEA data. **a**, **b** Positions of the NFC extremities (South and North) obtained by random sampling within the determined cones and calculation of the corresponding intersection points. The (x;z) correlation arises from the observation direction. **c**, **d** Length and slope obtained from this sampling, with fits of these distribution giving mean value and error bars. **e**–**g** Results of the *χ*^2^ method to determine the East-West shift *Y*, the height *H* and the width *W* of the NFC by each of the five instruments: each point corresponds to a simulation with a different *Y*, *H* or *W*. The *χ*^2^ is computed on a 1D histogram of the difference between data and simulation. The horizontal colored lines show the statistical error bars obtained for each instrument to display the data compatibility. **h** *χ*^2^ dependence as a function of *Y* was obtained by applying the same analysis to pseudo-data generated with the full simulation. **i** Integrated density distribution (in g/cm^3^) obtained between *Z* = 19.5 m and *Z* = 23.5 m using the SART algorithm as described in the text. The Chevron shape is correctly reconstructed as well as the NFC position, without any geometry input.

The combination of all the cones allows to determine the extremity positions by calculating the intersection points taking into account the width of each cone. The error bars on these positions were determined by Monte Carlo, randomly choosing a direction within each cone (with Gaussian statistics) and calculating the corresponding intersection points, see Fig. 8a, b. The resulting distributions of these point coordinates directly determine the length of the NFC, i.e. (9.23 ± 0.48) m (Fig. 8c), with a slope compatible with 0, (−1.9 ${}_{-4.7}^{+7.2}$)° (Fig. 8d) and located on its North extremity at *Z* = (1.34 ± 0.53) m.

The East-West position *Y* of the void was accurately determined with a set of 15 simulations per instrument using a NFC at different *Y* from −8 m to −6 m. For each of these 15 × 5 simulations, a 2D histogram was formed with *tan*(*θ*_*y*_) ranging from the two determined extremities of the NFC and with a 0.5 width in *tan*(*θ*_*x*_) centered on the NFC. This 2D histogram was then integrated in *tan*(*θ*_*y*_) to form a 1D histogram in the *tan*(*θ*_*x*_) direction, whose reduced *χ*^2^ was calculated. Figure 8e shows these *χ*^2^ values as a function of *Y* for the 5 instruments. The obtained values exhibit a standard parabola shape and a fit around the minimum provides the most likely value of *Y* for each instrument. As can be seen, all the values are well compatible (see also Supplementary Table 1) and the minimum *χ*^2^ are relatively low, except for Charpak-1 whose data suffered from very localized noise. The statistical error bars are obtained by considering all *Y* values with values from ${\chi }_{min}^{2}$ and ${\chi }_{min}^{2}$+1. In addition to the statistical error, a systematic error was taken into account from the uncertainty on the *γ* angle of each instrument. A global systematics of 10 cm was also added as the estimated precision of the 3D geometry model. All in all, one obtains: *Y* = (−0.07 ± 0.04 (stat.+syst.) ± 0.10 (syst. 3D model)) m = (−0.07 ± 0.11) m. The relative shift of the NFC compared to the DC is thus only (−7 ± 4) cm.

The same exercise was performed to determine the height *H* of the NFC. The resulting *χ*^2^ values are shown in Fig. 8f and are again well compatible (see Supplementary Table 1). In this case, an additional set of 15 simulations per instrument was generated with a density of 2.0 g/cm^3^ instead of 2.2  g/cm^3^ in order to estimate the systematics associated with the density uncertainty. A 10 cm difference was observed on the mean value, leading to the following result: *H* = (1.85 ± 0.07 (stat.) ± 0.10 (syst.)) m = (1.85 ± 0.12) m.

The width of the NFC was then estimated with the same method. The results are shown in Fig. 8g and Supplementary Table 1. For each instrument, a systematic coming from the *Z* position uncertainty of the NFC (estimated to 0.5 m) should be taken into account, yieding values from 5 to 8 cm depending on the distance of the telescopes to the NFC. This systematic effect being fully correlated for all the telescopes, a conservative, global systematics of 8 cm was applied, leading to: *W* = (1.87 ± 0.08 (stat.) ± 0.08 (syst.)) m = (1.87 ± 0.11) m.

The same analysis was performed with pseudo-data generated from Joliot position using the full simulation program but with scans from the fast version. The goal was to check the absence of bias of this analysis, by introducing a NFC and reconstructing its position and dimensions. As illustrated on Fig. 8h, the NFC parameters were correctly reconstructed. More specifically, the reconstructed East-West position was found to be (−0.08 ± 0.10) m (for a simulated position at −0.12 m), the height (1.95 ± 0.22) m (for a simulated value at 2.00 m), and the width (1.47 ± 0.21) m (simulated at 1.50 m).

While the above analysis relies on a somehow arbitrary assumption that the NFC has a parallelepiped shape, this assumption is well supported by the comparison with the simulation implementing the fitted NFC, where the local excesses attributed to the NFC largely disappear (Fig. 7h–j). However, it’s worth mentioning that other shapes are compatible with data, and the chosen parallelepiped only represents the simplest possible form. If the real shape is more complex (e.g. with a Chevron structure on the ceiling), the obtained *mean* dimensions wouldn’t change. Last but not least, a southwards continuation of the NFC with a smaller transverse section cannot be ruled out with the current statistics, but simulations showed that it should then be smaller than 1.0 m × 1.0 m to remain unseen by our instruments.

All the analyses shown up to now rely on the explicit comparison with a 3D geometric model of the pyramid to prove the existence of the NFC. Though the observed muon excess cannot reasonably be attributed to any bias from this model, it is however interesting to try imaging the NFC independently on any geometry input, only by combining the 2D muographies in a 3D picture like in medical imaging. We thus implemented an iterative, SART algorithm^22^ first applied to images obtained with absorbed muons^23^ and more recently to images obtained with transmitted ones^24,25^ (Methods). In spite of the small number of projections and the enormous size of the object, this algorithm correctly localized the NFC (Fig. 8i). The precision reached in this case is evidently worse than the 2D analysis (as the data are not integrated along the observation direction anymore), but this direct reconstruction does not suffer from any systematic nor potential errors from the 3D model, the geometry of the pyramid becoming an output in this analysis.

## Discussion

The results presented in this work were obtained by two teams using different muon detectors and independent analyses. As can be seen in Table 1, all the NFC parameter estimates are well compatible within error bars, and the previously discovered NFC was measured with excellent sensitivity. North end of the corridor starts at 0.8 m behind the Chevron and ends at 9.1 m southward. A horizontal corridor is favored, with a transverse section around 2.0 m × 2.0 m. More complex shapes than a simple paralleliped are possible, but with the same mean height and width. To our knowledge, this study is the first characterization of the position and dimensions of a void detected by cosmic-ray muons with a sensitivity of a few centimeters only. On the archaeological side, the discovery of a void located behind the Chevron and having a larger cross-section than the corridors connecting the inner structures of the pyramid may be decisive in approaching the role of this Chevron. Last but not least, the NFC does not seem to connect to the SP-BV, though a smaller corridor of <1.0 m between these two structures cannot be completely ruled out from these measurements.

**Table 1:** Summary of the NFC characteristics

Parameter	Nagoya estimate	CEA estimate
Width *W* (m)	2.02 ± 0.06	1.87 ± 0.11
Height *H* (m)	2.18 ± 0.17	1.86 ± 0.12
Length *L* (m)	9.06 ± 0.07	9.23 ± 0.48
North-South *X* (m)	0.84 ± 0.05	0.45 ± 0.22
East-West *Y* (m)	0.03 ± 0.04	−0.07 ± 0.11
Altitude *Z* (m)	0.72 ± 0.13	1.34 ± 0.53
Slope *α* (°)	−0.3 ± 1.5	−1.9 ${}_{-4.7}^{+7.2}$

Dimensions, positions, and orientation were obtained independently by Nagoya and CEA analyses. The East-West shift is taken from the descending corridor. X is positive for South. Y is positive for West.

## Methods

### Installation of nuclear emulsion films

A nuclear emulsion film which has an area of 25 cm × 30 cm was manufactured, and packed with vacuum in an aluminum laminated envelope for protecting light, dust and keeping humidity of films for portability at Nagoya University^6,9,16^. Nuclear emulsion films and detector components to fix them on installation positions were separately brought into Khufu’s Pyramid, they were assembled at installation places and the precise location of the installation has been surveyed. By stacking two or more films at the beginning of the observation and selecting the tracks that penetrate those nuclear emulsion films during the observation period, the noise tracks accumulated in the nuclear emulsion films and the muons recorded during the observation were discriminated. The nuclear emulsion films were changed every several months. The period was decided by consideration of the latent image fading characteristic of them and noise track accumulation by natural radiation from the component of kind of stones that construct the Pyramid. The observation by nuclear emulsion film was started in 2015 and continued by changing the kind of nuclear emulsion films and configurations. However the change of nuclear emulsion films installed in November 2019 could not be conducted due to COVID-19 pandemic. Observation conducted in 2019 was used for this analysis by consideration of performance of muon detection efficiency and configuration of detectors. Two types of detectors were used: A detector with a total thickness of 1 cm or less, consisting of two aluminum plates of 380 mm × 310 mm × 2 or 5 mm sandwiching two or more stacked nuclear emulsion films (Fig. 2e) and another detector with a total thickness of 5 cm or less, consisting of two aluminum plates of 870 mm × 300 mm × 17 mm sandwiching three sets of two or more stacked nuclear emulsion films (Fig. 2c, d). The first, smaller type has an effective detection area of 0.075 cm^2^ defined by the size of the nuclear emulsion films and could be installed in a narrow cavity on side wall of the al-Ma’mun Corridor (MC). The second, larger type has an effective detection area of 0.225 cm^2^ which is the area of 3 sets of nuclear emulsion films and could be installed efficiently in the descending corridor (DC), respectively. The names of the detectors in this analysis are EM followed by the main number from 1 to 7, which is determined by the location of the detector. The detectors with multiple detectors installed at the same location were given alphabetical sub-IDs. Specifically, EM2, in which three aluminum honeycomb detectors were installed in the DC in a north-south direction, was named EM2N, EM2C, and EM2S for each aluminum honeycomb detector, starting from the north side. As for EM6, which consists of tilted aluminum plate detectors installed on top of horizontally placed aluminum plate detectors installed in the MC, the horizontal detectors are named EM6H and the tilted detectors are named EM6T (Fig. 2b).

### Chemical development and read out of nuclear emulsion films

The chemical development of nuclear emulsion films was conducted in the dark room constructed in the Great Egyptian Museum Convention Center (GEM-CC) and the films were then brought to Nagoya University for analysis. They were read out by the nuclear emulsion scanning system called Hyper Track Selector (HTS) at Nagoya University to reconstruct the tracks which penetrated through two piled emulsions during the data taking. Finally, muon tracks were selected with high purity by eliminating low energy tracks, e.g. electrons, from all the reconstructed tracks.

### 3D model of the pyramid and position accuracy of detectors for nuclear emulsion films in the model

The 3D model, which includes the DC, the MC and part of the area on the north face of the Khufu’s Pyramid containing the Chevron, was obtained by Cairo University, HIP and Emissive (Figs. 2b and 3a). During the scanning of the DC and the MC to create the 3D model, the nuclear emulsion films in place at the time were also captured simultaneously. The origin of the 3D model for the analysis of nuclear emulsion films was defined as the north face of the Chevron for *x*-axis, the east-west center of the Chevron for y-axis, and the feature point of the Chevron where the lower cross point of the huge gabled limestone beams for *z*-axis, respectively (Fig. 3a). The positions of the detectors in the 3D model were determined with an accuracy of 3 cm in the north-south direction and 2 cm in the east-west direction for the DC and 5 cm for the MC, based on the survey records during installation of detectors and the measurements of the positions of the scanned detectors by pointing the feature points of the detectors in the 3D model.

### Simulation method for nuclear emulsion films

Two kinds of simulators were developed to estimate value of the muon flux observed at each detector position using the Geant4 (version 10.4.2) Monte Carlo simulation toolkit^10–12^. The first method is to analytically calculate the number of muons to be observed at the detector considered as a point without area from the density length (g/cm^2^) defined as the path length (cm) multiplied by the density (g/cm^3^) in each direction, which is obtained from the detector position in the 3D model, and is referred to as a simplified fast simulation. The second method is a Monte Carlo simulation that incorporates a muon generator that takes into account the muon energy distribution, angular distribution, irradiation region and detector area. The 3D model was converted to GDML format, which is commonly used in Geant4, for the simulation. The simple simulation takes a few seconds to calculate the angular distribution of the muon flux for a single condition. In order to determine the conditions for the simulation, the simplified fast simulation were performed under a combination of different densities of stones made of the Pyramid (1.8 g/cm^3^ to 2.6 g/cm^3^ at 0.2 g/cm^3^ intervals) and nine different muon flux models (Miyake^26^ (integration and differentiation), Jockich^27^ (integration and differentiation), Bogdanava^17^, Reyna^18^, Tang^19^, Guan^13^, and Sato^28,29^). The angular distributions of each simulation and observation were compared to each other in the region excluding the NFC. Among the evaluated conditions, the simulation result using the Guan model with a pyramid density of 2.2 g/cm^3^ provided the best agreement with the data, so the simulations were performed in these conditions. This result can be interpreted as the fact that the Guan equation best describes the angular dependence of the energy distribution in the low energy region among the compared models. Parameters for running simulation and resolution of 3D model were decided to achieve a reasonable speed of the Monte Carlo simulation as described below. The minimum energies of muon to be exposed in the simulation were set to 0.2 GeV for EM1, 0.5 GeV for EM2, 1 GeV for EM7, 3 GeV for EM5, EM6 and EM7, and 5 GeV for EM3 and EM4, by considering minimum energy of muons passed through pyramid to each detectors. Muons were irradiated in the angle range of 0–60° (−1.73 ≦ $\tan \theta$ ≦ 1.73) from the axis perpendicular to the detector surface and in an area slightly larger than the detector area, by taking into account the effect of scattering of muons while passing through the pyramid and angular range for analysis to avoid inefficiency of muon exposure. Electromagnetic process and decay process in terms of physical interactions are computed and only muons are traced in the simulation. The Monte Carlo simulations under these conditions took about a day to perform on a PC configured with an AMD Ryzen Threadripper 3970X 3.7 GHz 32 cores/64 threads, which is equivalent to 100 days of calculations in terms of real-world observation time.

### Data correction of the nuclear emulsion films installed in the descending corridor

The analyzed data are from detectors installed at four locations at the DC on five separate occasions (Feb. to Apr., Apr. to Jun., Jun. to Jul., Jul. to Sep., Sep. to Oct.) from February to October 2019. The reconstructed muon tracks were integrated for four observations (equivalent to 172 days) for EM1, five observations (211 days) for EM2 and EM3, and two observations (79 days) for EM4.

### Estimation of path length by the analysis of the nuclear emulsion films installed in the descending corridor

The density length was calculated by using the formula of muon flux given by Guan^13^ and the relationship between muon energy and flight length in the case of the limestone density assumed 2.2 g/cm^3^ based on the table given by GROOM et al.^30^. The path length was calculated by dividing the density length by the density of 2.2 g/cm^3^ (Fig. 3b, c).

### Initial analysis of the NFC by the nuclear emulsion films installed in the descending corridor

Distributions of the path length with the difference between observed data and simulation, which corresponds to length of the NFC along the line of sight in the axial direction of $\tan {\theta }_{y}$, are shown in Fig. 3c. The distributions of the path length by all detectors are consistent and it’s value is ~2 m. The directions corresponding to the northern and southern ends of the NFC were identified if they were within the observed field of view. By checking the range where they intersect in three dimensions, we estimated the angular range and location where the NFC exists (Fig. 3d). The NFC is located in the angular region of $\tan {\theta }_{y}$ = −0.1125 or less by EM1, $\tan {\theta }_{y}$ = −0.0125, 0.0375 and 0.0875 or less by EM2N, EM2C and EM2S, and between of 0.0125 ≦ $\tan {\theta }_{y}$ < 0.6625 by EM3 and 0.1125 ≦ $\tan {\theta }_{y}$ < 0.7125 by EM4, in $\tan {\theta }_{y}$ coordinate, respectively. The estimated location in height is above 0 m in z.

### *χ*^2^ analysis and parameter estimation accuracy by the nuclear emulsion films installed in the descending corridor

The evaluation area of reduced *χ*^2^ were −0.05 ≦ $\tan {\theta }_{x}$ < 0.05, −0.2 ≦ $\tan {\theta }_{y}$ < 0.2 for X, −0.15 ≦ $\tan {\theta }_{x}$ < 0.15, 0.1 ≦ $\tan {\theta }_{y}$ < 0.4 for Y and W, −0.05 ≦ $\tan {\theta }_{x}$ < 0.05, 0.4 ≦ $\tan {\theta }_{y}$ < 0.8 for Z, −0.05 ≦ $\tan {\theta }_{x}$ < 0.05, 0.1 ≦ $\tan {\theta }_{y}$ < 0.4 for H, and −0.05 ≦ $\tan {\theta }_{x}$ < 0.05, −0.2 ≦ $\tan {\theta }_{y}$ < 0.1 for L. Simulations for the *χ*^2^ evaluation corresponded to 500 days of observations for X, 400 days for Y, W and L, 800 days for Z and 200 days for H. For the evaluation of X (location of the northern extremity), the data and simulations were normalized for a region of −0.3 ≦ $\tan {\theta }_{x}$ < −0.2, 0.2 ≦ $\tan {\theta }_{x}$ < 0.3 for each slice in $\tan {\theta }_{y}$ divided by a width of $\tan \theta$ = 0.025 for EM2. For the evaluation of Z, W and Y, the data and simulations were normalized for a region of −0.2 ≦ $\tan {\theta }_{x}$ < −0.15, 0.15 ≦ $\tan {\theta }_{x}$ < 0.2 for each slice in $\tan {\theta }_{y}$ divided by a width of $\tan \theta$ = 0.025 to suppress the influence of small excess due to sub-structure, for EM3. For the evaluation of H, the data and simulations were normalized for a region of −0.5 ≦ $\tan {\theta }_{x}$ < −0.2, 0.2 ≦ $\tan {\theta }_{x}$ < 0.5 for each slice in $\tan {\theta }_{y}$ divided by a width of $\tan \theta$ = 0.025 for EM3. Assuming a void model of a single horizontal rectangular solid, the value of *χ*^2^/ndf was derived by comparing observations with simulations in which the numerical value of one of the parameters under evaluation was changed. By fitting five values including the minimum value of *χ*^2^/ndf in the evaluated parameters with a quadratic function, a value of the parameter with the minimum *χ*^2^/ndf was obtained, and the statistical error of the decision accuracy was determined (Fig. 3e). The validity of the *χ*^2^ analysis was evaluated using the simulation based on Geant4 and its accuracy was estimated to be a few cm, except for *Z*. Since the value of *Z* was evaluated by normalizing to the region containing the sub-structure, the small excess of muons due to it would cause a systematic shift on the *Z* position of the main structure. The effect was estimated by a simulation with the sub-structures and *Z* was estimated to be 0.45 m ± 0.05 m higher than the input location. This systematic shift was included in the parameter estimate. Furthermore, a 10% variation in pyramid density, assumed to be 2.2 g/cm^3^ in this analysis, yielded a systematic error of 0.16 m in H. The effect of secondary electrons generated when cosmic rays penetrate the stone material of the pyramid was estimated to be up to 1.5% on H. Other systematic errors were estimated to take into account the 3D model accuracy, the detector positioning in the 3D model, the accuracy of installation of the detectors, and propagation of error by the evaluation order. These evaluations lead to the following estimates: *X* = 0.84 ± 0.02(stat) ± 0.05(syst) m, *W* = 2.02 ± 0.05(stat) ± 0.03(syst) m, *H* = 2.18 ± 0.04(stat) ± 0.16(syst) m, *L* = 9.06 ± 0.05(stat) ± 0.05(syst) m, *Z* = 0.72 ± 0.08(stat) ± 0.11(syst) m and *Y* = 0.03 ± 0.02(stat) ± 0.03(syst) m. The systematic error on H includes a density variation of 10%.

### Estimation of the southward stopping position of NFC and its cross-sectional shape

An estimation was conducted to determine how far the NFC continues to the south (Fig. 3c). Simulations for two void models with cross sections of 2 m × 2 m and 1 m × 1 m for EM3 were performed, and it was estimated that those voids can be detected significantly beyond $\tan {\theta }_{y}$ = 0.0 to about −0.3. Therefore, there is no structure with a cross section larger than 1 m × 1 m in the south, further than the direction where the NFC appears to stop.

### Data correction of the nuclear emulsion films installed in the al-Ma’mun Corridor

The analyzed data were obtained from the detectors installed at three locations in the al-Ma’mun corridor on six separate terms (Feb. to Apr., Apr. to Jun., Jun. to Jul., Jul. to Sep., Sep. to Oct., Oct. to Nov.) from February to October 2019. The reconstructed muon tracks were integrated for six observations (equivalent to 272 days) for all detectors (EM5, EM6H, EM6T, and EM7).

### Data integration of the nuclear emulsion films installed in the al-Ma’mun corridor

A systematic deviation was seen in the comparison of angular distribution of the muon flux between the observation and simulation with the initial azimuth angle. This deviation is likely due to a difference between the azimuth angle measured by the analog compass during the survey and the actual value from the compass near the magnetic material used to construct the detectors. Therefore, a comparison was conducted between the observation and the simulations by simplified fast simulation with azimuth angle of the detector varying in 0.5° increments at a density of 2.2 g/cm^3^, in order to find the angle providing the best angular distribution agreement.

### Calibration of the azimuthal angle of the nuclear emulsion films installed in the al-Ma’mun corridor

The agreement between observed and simulated angular distribution was evaluated from the standard deviation of the differences of the value in each bin in the angular range including the structures around the Chevron because of the regions with large changes in the undulations of the shape are effective to determine the direction of the detectors. The regression curve was calculated using the azimuthal dependence of the standard deviation in the region evaluated in 0.5° increments, and the azimuthal angle with the minimum standard deviation in 0.1° increments was determined.

### Projection analysis by the nuclear emulsion films installed in the al-Ma’mun corridor

The distribution of differences between observation and simulation of path length corresponds to the superimposed distribution of the structure of the NFC (Fig. 4a, c). The direction distribution of maximum of path length differences is considered to be indicated the distribution of center of the NFC in the case of simple structure. The location of the center of the NFC in height was estimated by projecting the direction of maximum direction of path length differences, which corresponds to the NFC, to the vertical plane on the center axis of the DC, because the location of the NFC is considered to be just above the DC from the results of the detectors installed in the DC. The projected direction was determined by making the histogram of a cross section of the angular distribution, which is perpendicular to the longitudinal direction (north-south direction) of the NFC, and fitting each histogram by a Gaussian function to obtain the center value (Fig. 4c). Figure 4d shows the intersection of a vertical plane passing through the central axis of the DC and a line extending towards the plane in the direction of the central value of the histogram fitted starting from the detector position. The location of the centre of the NFC was determined to be 2.0 ± 0.5 m using a range of −2 to −8 m in the x corresponding to north-south direction by eliminating the area which included the outer part of the NFC to avoid the effect of pointing in the wrong direction (Fig. 5b). Errors were estimated by standard deviation of distribution in height and they are reasonable values considering the accuracy of determination of the detector position in the 3D model, systematic errors of the projection method, and errors due to fitting. The slope of the NFC in north-south direction was calculated by linear approximation using the least squares method for the same range of estimation of location in height, and was estimated to be (−0.3 ± 1.5)°. The length of the NFC was estimated to be ~10 m in maximum and it appears to stop at the south side from starting at just behind the Chevron.

### Installation and operation of the CEA telescopes

The 3 telescopes were installed from Oct. 19th to 22nd, 2019. After sufficient gas flushing, the acquisition started on Oct. 19th on Degennes, and Oct. 25th on Joliot and Charpak. All the telescopes showed excellent and stable performance (see Supplementary Fig. 1). They all stopped on March 13th 2020 after an electricity cut due to a storm. Charpak was restarted on March 18th at a reduced gas flow, and definitely stopped on April 6th following the COVID-19 pandemic. All in all, Degennes and Joliot accumulated about 140 days of stable data, and Charpak around 158 days, yielding respectively 29.8, 13.0 and 73.4 millions of good muons.

### CEA simulation

The 3D CAD model used in the simulations was obtained by Cairo University, HIP and Emissive, and is therefore identical to the one used by Nagoya. To speed up the simulation time, parametrizations of all the important phenomena (energy loss, straggling and multiple scattering) were derived from Geant4, so that muons can be simulated without the whole, time consuming Geant4 propagation in the pyramid. This fast Monte Carlo used a 2D distance map generated for each telescope position. While the full Geant4 simulation takes about 50 days to produce the equivalent of 100 days of data taking, the fast simulation requires only 3 min on a standard laptop. The 2D muographies obtained from the Joliot position by the fast and the full Monte Carlo simulations (using only muons and no secondary electrons) were compared and showed no significant difference (see Supplementary Fig. 2).

### Analysis of the telescopes data

The 2D raw muographies were obtained for each instrument from the reconstruction of muon trajectories requiring a signal in at least 3 detectors out of 4. Thanks to this redundancy, the small detector inefficiency (<5%) can be safely neglected. A precise comparison with the simulation then required the determination of telescope orientations, defined by Euler angles. The rotation around vertical axis (*α*) was very precisely fixed by aligning the telescopes with the DC and AC corridors, allowing for the telescopes to be aligned in the North-South axis with a precision of about 0.2° (2 cm accuracy over the 60 cm telescope length). The 2 other angles *β* (rotation around East-West axis) and *γ* (rotation around North-South axis) were measured with a 1° accuracy. For each of the 5 instruments, a set of 15 simulations were performed at different values of *β* and 15 others at different values of *γ*. For each of them, the 2D muography ratio between data and simulation was computed. For the scan in *β* (resp. *γ*), a 1D histogram was then formed along the *tan*(*θ*_*y*_) (resp. *tan*(*θ*_*x*_)) axis by integrating in a wide range of *tan*(*θ*_*x*_) (resp. *tan*(*θ*_*y*_)). In both cases, the NFC zone was excluded from the scanned region not to bias the result. The *γ* scan is illustrated in Supplementary Fig. 3 for Degennes telescope. The *χ*^2^ of each 1D histogram is then calculated as a function of *β* and *γ* to determine the best value. Such a comparison allows for a determination of these angles with a 0.2 to 0.4° accuracy.

Once the position and orientation of all telescopes are precisely adjusted, the comparison between data and simulation in the NFC region provides its extremities as seen by each instrument. Supplementary Fig. 4 shows an example of the horizontal slicing performed on the 2D muography of Degennes-Q2. The North extremity is thus observed at *tan*(*θ*_*y*_)= 0.49 ± 0.01, the same exercise at the South side leading to *tan*(*θ*_*y*_)= 0.11 ± 0.01.

### 3D reconstruction with SART algorithm

An alternative way to reconstruct a structure like the NFC is to combine the 2D muographies to obtain a 3D tomographic image. This method is routinely used in medical imaging, but with many more projections and a lot more of statistics. Following a first implementation of the SART algorithm in the special mode of absorption muography^23^, a procedure was developed to apply it to all the CEA data taken inside the pyramid (representing 11 sets of data). The volume to image is splitted into *N*_*v*_ 3D pixels (or *voxels*), and each of the *n* data set *i* (a dataset is a 2D muography) is separated in ${n}_{m}^{i}$ independent measurements. The aim is then to solve the following equation:

1 $${{{{{{{\boldsymbol{[A]}}}}}}}}{{{{{{{\boldsymbol{\rho }}}}}}}}={{{{{{{\boldsymbol{O}}}}}}}},$$

where ***ρ*** is the unknown density *N*_*v*_-vector, representing the density in each voxel, ***O*** is the opacity vector, of size *N*_*m*_=${\sum }_{i}{n}_{m}^{i}$, and ***[A]*** is the distance matrix whose element (*k*,*l*) contains the mean distance traveled in the *l*^*th*^ voxel by muons from the *k*^*th*^ opacity measurement. This distance matrix can be calculated once the voxels and the data splitting have been chosen. But on the contrary to the absorption mode^23^, the opacity cannot not be directly obtained from the data alone. An open-sky simulation at each telescope position should first be performed in order to get the muon flux in each ${n}_{m}^{i}$ measurement, and therefore the muon absorption factor. An additional set of Geant4-based simulations are then generated using different thicknesses of materials, to parametrize the relation between the opacity and the muon absorption factor. The muon energy distribution being dependent on the azimutal angle, this parametrization is angle dependent^24^.

Once the opacity vector is determined, the SART algorithm iteratively updates the *v*^*th*^ voxel density by adding the following value at iteration *n* + 1:

2 $$\Delta {\rho }_{v}^{n+1}=\frac{\mathop{\sum }\nolimits_{m=1}^{{N}_{m}}{w}_{m}\frac{{O}_{m}-{O}_{m}^{n}}{{\overrightarrow{{A}_{m}}}^{2}}}{\mathop{\sum }\nolimits_{m=1}^{{N}_{m}}{w}_{m}}\times {A}_{m,v},$$

with ${O}_{m}^{n}$ being defined as:

3 $${O}_{m}^{n}=\mathop{\sum }\limits_{v=1}^{{N}_{v}}{O}_{m,\;v}{\rho }_{v}^{n},$$

and *w*_*m*_ is a weight coming from the statistics in the *m*^*th*^ measurement.

The volume to image was chosen to be a parallelepiped of size 230 × 230 × 140 m^3^ thus containing the whole pyramid, with 1 × 1 × 1 m^3^ voxels. The 11 data sets were each splitted in 100 × 100 measurements, resulting in a distance matrix with about 814 billion elements. The density vector is initiated using a perfect, full pyramid of density 2.0 g/cm^3^, thus without any inside or outside structures. The result shown in Fig. 8i is obtained after the equivalent of 150,000 iterations, and represents the density distribution in a slice between *z* = 19.5 and 23.5 m above the ground in the vicinity of the Chevron. In spite of the enormous size of the system to be solved, the algorithm correctly identifies a strong under-density and the overall shape of the Chevron zone with its East-West asymetry, as well as an under-density corresponding to the NFC. Because of the limited resolution in the *z* axis, the under-density is diffused over about 5 m high, and the NFC height cannot be fully resolved by this technique. Though less precise than the 2D triangulation performed above, this procedure does not require any precise geometrical model of the object to image, as this geometry becomes an output of the analysis.

## Supplementary information

Supplementary Information

## Data Availability

The raw data that support the findings in this study are available from the corresponding authors. Please note that the data sharing should be approved by the Egyptian Ministry of Antiquities. The project coordinator (Prof. H. Helal) is responsible for the communication with the Ministry regarding this point.

## Code availability

The analysis code that supports the findings in this study are available on reasonable requests from the corresponding authors.
