# Supplementary material for: Precise characterization of a corridor-shaped structure in Khufu’s Pyramid by observation of cosmic-ray muons
Source: Nat Commun. 2023 Mar 2;14:1144. doi: 10.1038/s41467-023-36351-0 (PMC9981702; doi:10.1038/s41467-023-36351-0)
Supplement: Supplementary file 1 — Supplementary Information [file 41467_2023_36351_MOESM1_ESM.pdf]

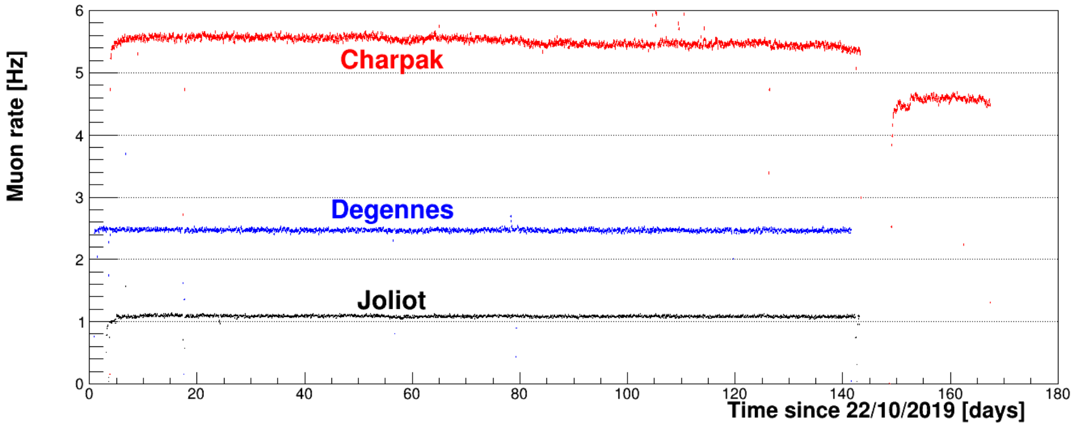

Supplementary Figure 1 : Stability of data taking with CEA telescopes. Evolution of the muon rate recorded on the 3 telescopes during the data taking. The last part of the Charpak acquisition was done with a reduced gas flow.

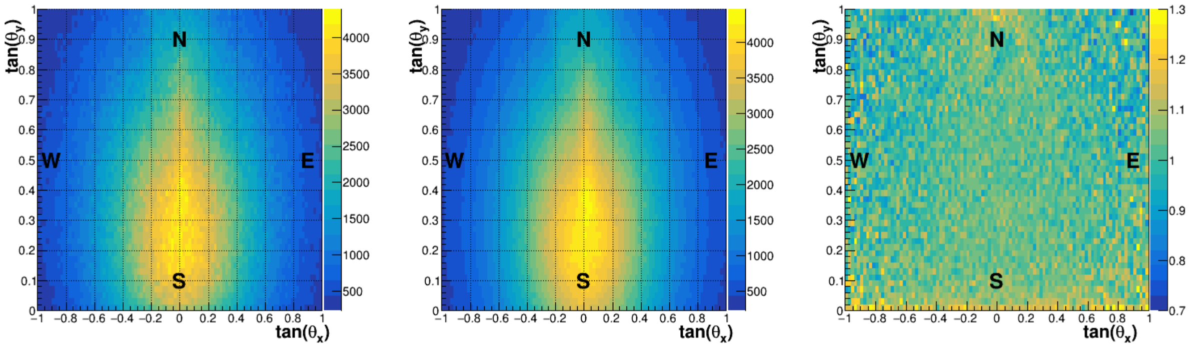

Supplementary Figure 2 : Comparison of the full and fast simulations. Full (left) and fast (middle) simulation of Joliot data, and the corresponding ratio (right). The comparison was limited to tan(θ_y) > 0 to speed up the full simulation generation. No significant difference is observed, while the second version is more than 20,000 times faster.

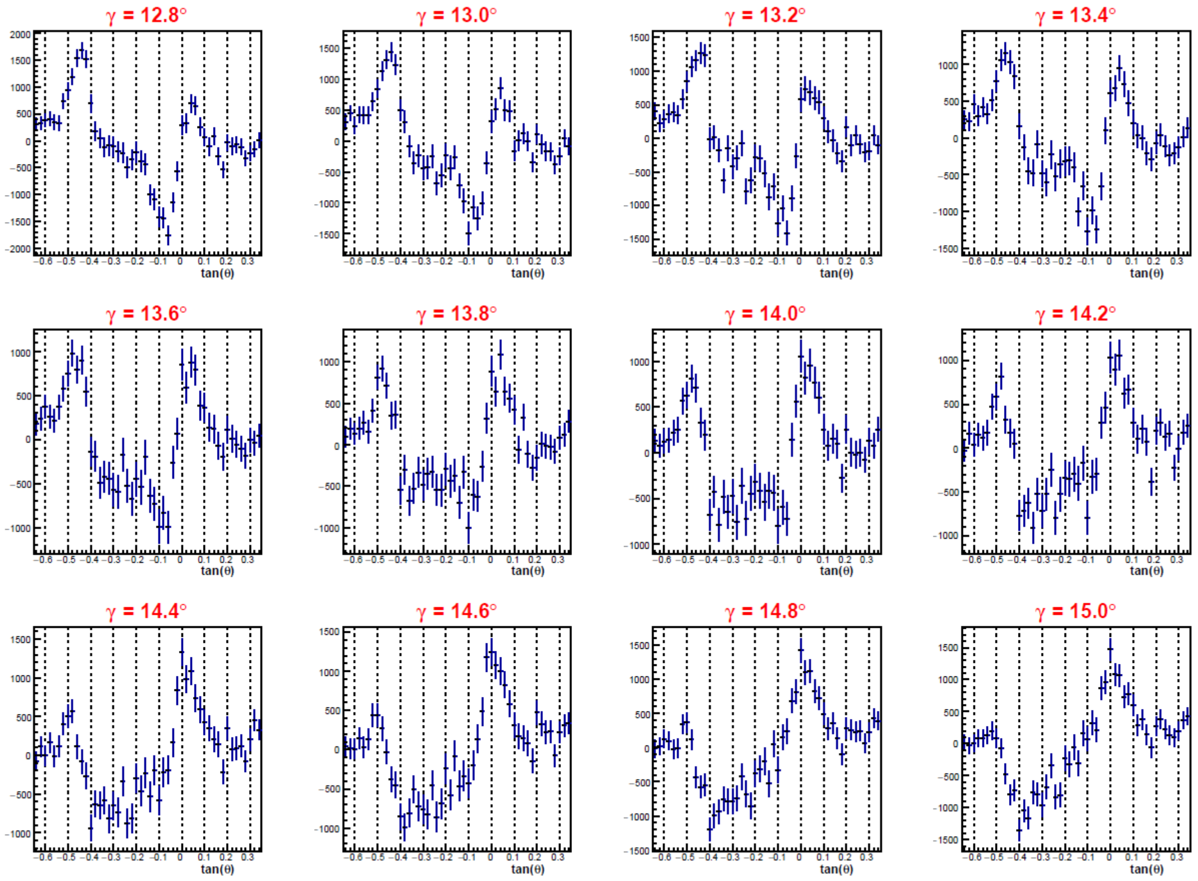

Supplementary Figure 3 : Software determination of the Degennes-Q2 telescope orientation. Ratio between Degennes-Q2 data and a set of simulations obtained with different γ values, integrated in tan(θ_y) in the vicinity of the Chevron zone. As stated in the paper, the agreement is limited because of the higher density of the Chevron, but the shape of the histograms allow for γ determination, in this case $(13.8 \pm 0.4)°$.

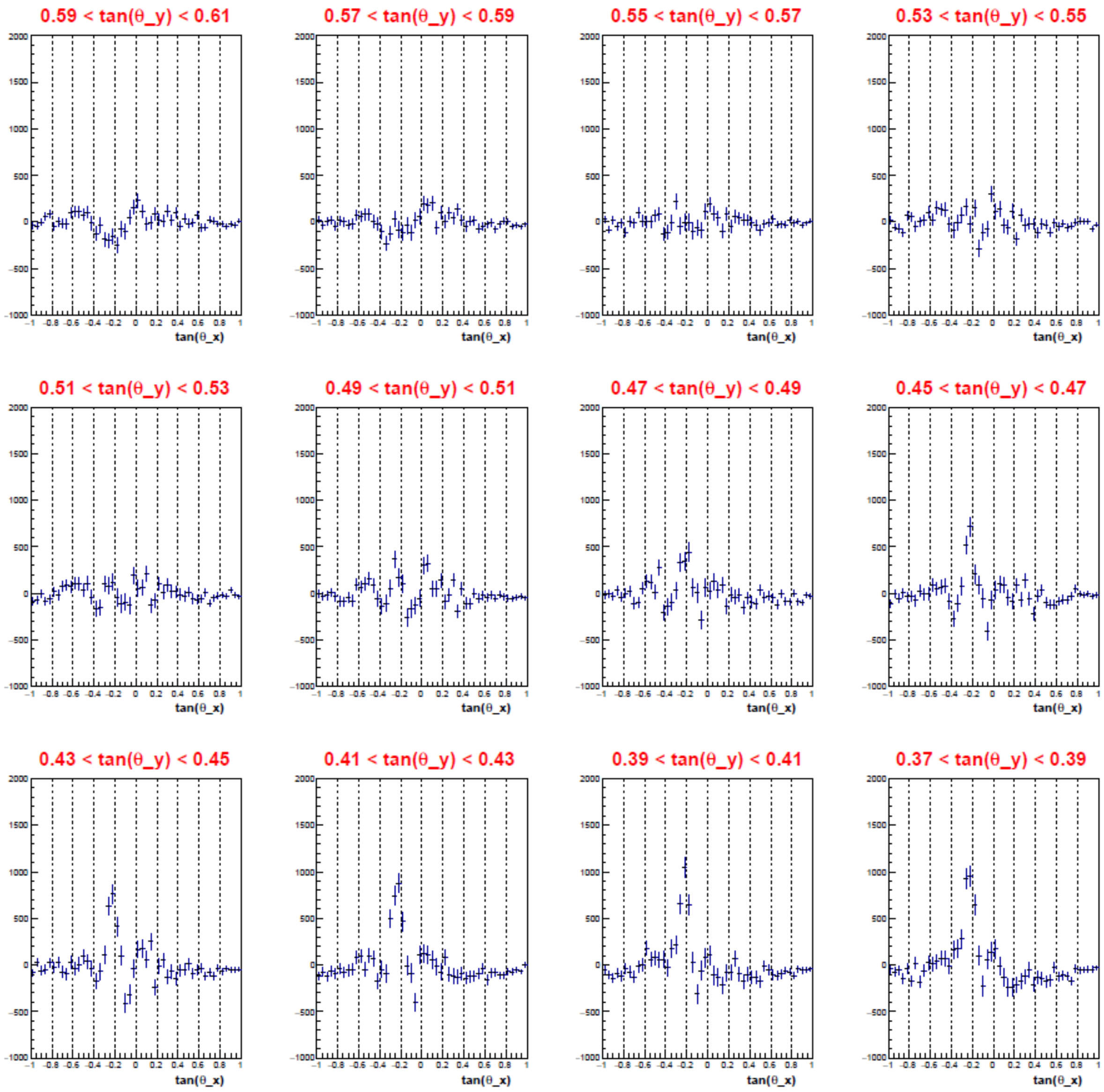

Supplementary Figure 4 : Determination of the NFC extremity with Degennes-Q2 telescope. Ratio between Degennes-Q2 data and simulation, obtained by slicing the 2D muographies in tan(θ_x) direction in bands of tan(θ_y) of 0.02 thickness (determined by the available statistics). The slices show the appearance of the NFC (North extremity) at tan(θ_y) = 0.49 $\pm$ 0.01 and tan(θ_x) around -0.22.

Supplementary Table 1 : NFC dimensions and positions independently obtained for each of the 5 CEA instruments (statistical error only).

Instrument	Y (m)	H (m)	W (m)
Degennes-1	0.01 $\pm$ 0.09	1.94 $\pm$0.21	2.29 $\pm$0.29
Degennes-2	0.11 $\pm$0.09	2.02 $\pm$0.19	2.13 $\pm$0.24
Joliot-1	-0.18 $\pm$0.09	1.78 $\pm$0.20	2.10 $\pm$0.28
Charpak-1	-0.03 $\pm$0.04	2.02 $\pm$0.12	1.73 $\pm$0.12
Charpak-2	-0.16 $\pm$0.04	1.66 $\pm$0.11	1.82 $\pm$0.14
